# Comparative effectiveness and underlying mechanisms of acupuncture approaches in IBS-D animal models: a protocol for systematic review and network meta-analysis

**DOI:** 10.1186/s13643-026-03162-5

**Published:** 2026-03-26

**Authors:** Meiyi Zhu, Lun Li, Di Zhang, Yuan Fang, Jia Huang, Jiahui Yang, Yongjian Liu, Xiumei Yan, Tian Zhang, Jiaqiang Jiang, Qinhong Zhang, Fan Jiang

**Affiliations:** 1 Jiangxi University of Chinese Medicine, Nanchang, China; 2 Affiliated Hospital of Jiangxi University of Chinese Medicine, Nanchang, China; 3 Heilongjiang University of Chinese Medicine, Harbin, China

**Keywords:** Irritable bowel syndrome, Acupuncture therapy, Diarrhea-predominant irritable bowel syndrome, Animal models, Network meta-analysis

## Abstract

Irritable bowel syndrome with predominant diarrhea (IBS-D) is a type of functional gastrointestinal disorder mainly marked by abdominal discomfort and frequent loose stools. Its complex pathogenesis involves multiple factors, such as low-grade intestinal inflammation, brain-gut axis dysregulation, and visceral hypersensitivity. Although acupuncture therapies show the potential to alleviate IBS-D symptoms, systematic evidence regarding their comparative efficacy and underlying mechanisms remains lacking.

**Methods** A systematic search will be conducted across eight major databases: CNKI, WanFang Data, VIP, CBM disc, PubMed, Embase, Web of Science, and CENTRAL. This search will aim to comprehensively collect randomized controlled animal studies on acupuncture therapies for IBS-D. Two independent researchers will extract and record basic study information, characteristics of experimental animals, intervention details, and outcome measures. The primary outcome to be evaluated will be the minimal volume threshold for the abdominal withdrawal reflex (AWR). Secondary outcome measures will include the loose stool rate and levels of biomarkers such as IL-1β, TNF-α, SP, and 5-HT. Data collected will be analyzed utilizing R 4.4.1 and Stata 17.0 software for frequentist network meta-analysis to compare the efficacy of various acupuncture treatment options in IBS-D animal models. Standardized mean differences (SMD) with 95% confidence intervals (CI) will be calculated as the primary effect measure. Treatment rankings will be generated using P-scores, the frequentist analogue of SUCRA. Additional analyses, such as subgroup analysis, sensitivity testing, and meta-regression will be performed. Risk of bias will be evaluated through the SYRCLE risk of bias tool. This study represents the first network meta-analysis integrating indirect and direct evidence from various acupuncture therapies in IBS-D animal models. It aims to provide high-level evidence for optimizing acupuncture therapy selection and clarifying its mechanisms. The network meta-analysis will generate robust preclinical evidence to identify the most effective acupuncture modalities for IBS-D and reveal their underlying mechanisms. The findings will guide future clinical trial design by informing the choice of optimal acupuncture strategies and relevant biological endpoints, thereby promoting the translation of preclinical evidence into clinical practice.

**Ethics and dissemination** Since this is a systematic review and meta-analysis, ethical approval is not deemed necessary.

**INPLASY registration number** INPLASY202470113.

**Supplementary Information:**

The online version contains supplementary material available at 10.1186/s13643-026-03162-5.

## Introduction

Irritable bowel syndrome with predominant diarrhea (IBS-D) is a prevalent functional gastrointestinal condition marked by repeated episodes of abdominal bloating, pain, discomfort, and loose stools [1]. These symptoms are often accompanied by variations in bowel patterns, including changes in stool texture or frequency [2]. The pathogenesis of IBS-D is complex, including factors such as brain-gut axis dysregulation, gut microbiota imbalance, chronic low-grade intestinal inflammation, intestinal barrier dysfunction, and psychological stress [3–6]. Increasing evidence indicates that core pathological processes involve disturbances in brain-gut axis function [7], neurotransmitter metabolism imbalance [8], and disrupted intestinal immune homeostasis [9, 10]. Current conventional treatments, including anti-diarrheal drugs, 5-HT receptor modulators, and low-FODMAP diets, offer some efficacy in symptom control [1]. However, these treatments are limited by significant individual variability in response, side effects (e.g., constipation, drowsiness), and poor long-term adherence, leading to a growing patient interest in alternative and complementary treatment options.

Acupuncture is an important part of alternative and complementary medicine and has attracted widespread attention due to its significant therapeutic effects and minimal side effects. Common acupuncture therapies include manual acupuncture, electroacupuncture, ear acupuncture, and moxibustion [11–14]. Although these therapies are all derived from traditional Chinese meridian theory, their specific operation methods and mechanisms of action are different, resulting in different therapeutic effects. For example, manual acupuncture stimulates acupoints through manual manipulation (twisting or lifting and inserting), activating somatic nerve reflexes, thereby affecting IBS-D [15]. Electroacupuncture may inhibit the release of SP by activating the vagal nerve-hypothalamic pathway [16]. Moxibustion may improve intestinal dysmotility by regulating the synthesis of 5-HT in enterochromaffin cells [17]. Ear acupuncture can regulate the intestinal immune microenvironment through vagal nerve-brain-gut interaction [18]. Given that pharmacological controls (e.g., loperamide, pinaverium bromide) are frequently employed as positive reference arms in preclinical acupuncture studies, the inclusion of such comparators in the network meta-analysis will strengthen network connectivity and provide clinically relevant context for interpreting acupuncture effects.

Animal experiments are highly valued because of their high controllability and repeatability and are an important basis for mechanism research. Researchers can objectively evaluate the biological effects of acupuncture therapy by measuring biochemical indicators (such as proinflammatory cytokines) and behavioral parameters (such as AWR threshold). However, most animal studies have focused on a single acupuncture therapy (e.g., electroacupuncture or moxibustion) or have only examined changes in specific biomarkers [10]. There is currently a lack of systematic and comprehensive comparisons of different acupuncture therapies on multiple indicators [11]. Network meta-analysis (NMA) provides an effective method to integrate research data on various acupuncture therapies in animal models of IBS-D. NMA integrates indirect and direct comparative evidence, allowing for a systematic assessment of how different acupuncture therapies improve IBS-D symptoms and affect related biomarkers. Through NMA, relative efficacy rankings can be analyzed to determine the advantages of the best acupuncture therapy and reveal the underlying mechanisms of different therapies.

The primary outcome evaluated in this research is the lowest volume threshold for AWR. This indicator is widely used in animal models to evaluate intestinal pain sensitivity [13, 19, 20], and directly reflects a core pathological feature of IBS-D. Secondary outcomes include inflammation-associated cytokines IL-1β and TNF-α, neuroregulatory peptides SP and 5-HT, and loose stool rate. Within the inflammatory pathway, TNF-α and IL-1β are key inflammation-related cytokines that exacerbate intestinal mucosal damage through the initiation of the NF-κB signaling pathway [3]. Elevated levels of these cytokines directly reflect the immune imbalance associated with low-grade intestinal inflammation in IBS-D [4]. In neural regulation, SP is a core neuropeptide mediating the transmission of visceral pain to the central nervous system, with elevated release significantly related to abdominal pain symptoms in IBS-D patients [6]. Conversely, 5-HT is a crucial neurotransmitter regulating gastrointestinal motility and visceral hypersensitivity [21], its abnormal metabolism is a major driver of diarrhea and motility disturbances [22]. Behaviorally, the loose stool rate serves as an objective measure of diarrhea severity, quantifying therapeutic efficacy and better assessing the regulatory effect of acupuncture interventions on intestinal transit function. Combining these outcome measures allows for a comprehensive reflection of the complex pathological mechanisms of IBS-D from multiple perspectives, including visceral hypersensitivity, inflammatory response, neurotransmitter regulation, and behavioral manifestations. This provides comprehensive data support for an in-depth exploration of the efficacy of acupuncture therapies.

Based on this background, this study aims to: (1) evaluate the comparative therapeutic effects of different acupuncture modalities in IBS-D animal models; (2) elucidate potential biological mechanisms underlying these effects; and (3) assess the influence of animal species, modeling methods, and treatment duration on outcomes. By systematically integrating direct and indirect evidence through network meta-analysis, this research will provide robust preclinical evidence to optimize acupuncture therapy selection and inform future mechanistic investigations.

## Methods

The protocol for this network meta-analysis and systematic review was registered on the INPLASY platform (registration number INPLASY202470113, https://inplasy.com/). This study strictly adhered to the Preferred Reporting Items for Systematic Reviews and Meta-Analyses Protocol (PRISMA-P) guidelines [23]. A completed PRISMA-P checklist is provided in Supplementary Material 1.

### Literature search

The timeframe for the search will be from the earliest available database records to April 30, 2025. The search end date of April 30, 2025, is a projected completion point for the literature search. Since the protocol was registered on July 23, 2024 (INPLASY202470113), this timeframe allows sufficient scope for comprehensive data collection and screening. If the review extends beyond this period, an updated search will be conducted within three months prior to full review submission to ensure inclusion of the most recent evidence. Any updates will be documented in the final systematic review, including the updated search date and number of additional studies identified.

Both Chinese databases (Wanfang Data, CNKI, VIP, and CBM(Chinese Biomedical Literature Database)) and English databases (Web of Science, PubMed, Embase, and CENTRAL) will be searched for studies on acupuncture therapy for IBS-D in animal models.

### Inclusion and exclusion criteria

The eligibility criteria for this review are defined according to the PICO framework as summarized in Table 1.

**Table 1:** PICO Framework for Study Eligibility

Element	Definition
Population	Rodent models (rats or mice) of IBS-D, induced by any method (chemical induction [e.g., acetic acid, senna], stress-based models [e.g., maternal separation, restraint stress, water avoidance stress], post-infection models, or combined methods), regardless of species, strain, sex, age, or weight
Intervention	Any acupuncture therapy based on meridian theory, including manual acupuncture, electroacupuncture, moxibustion, or combinations thereof, with no restrictions on acupoint selection, treatment frequency, or duration
Comparator	Sham acupuncture, blank/no treatment control, model-only control, or pharmacological control
Outcome	Primary: AWR minimal volume threshold. Secondary: loose stool rate, IL-1β, TNF-α, SP, 5-HT (see Section "Outcome measures" for definitions)

#### Inclusion criteria

(1) Study type: Randomized controlled Animal studies, published in either Chinese or English.(2) Subjects: All rodent models (rats or mice) of IBS-D, irrespective of model induction methodology, age, weight, or sex.(3) Interventions: Experimental groups: Acupuncture therapies based on meridian theory (e.g., manual acupuncture, electroacupuncture, moxibustion), with any combination of interventions, regardless of acupoint selection, duration, or treatment course. Control group: Sham acupuncture group, blank control group, model group, or pharmacological control group. Pharmacological controls (e.g., loperamide, pinaverium bromide) are included as comparator nodes—not as index interventions—to strengthen network connectivity and provide a clinically relevant reference for contextualizing acupuncture effects. The primary focus of the NMA remains the comparative effectiveness of different acupuncture modalities.

#### Exclusion criteria

(1) Duplicate publications. (2) Non-randomized controlled study designs. (3) Animals with other coexisting diseases or non-animal subjects. (4) Studies meeting inclusion criteria but with incomplete outcome data will be retained. (5) Studies whose eligibility cannot be determined due to insufficient information and where authors cannot be contacted for clarification. (6) Protocols, theses, reviews. (7) Literature not in Chinese or English.

Studies meeting the inclusion criteria but with incomplete outcome data will be retained in the systematic review. Where outcome data are missing or insufficiently reported for quantitative pooling, authors will be contacted to obtain the necessary data. If data remain unavailable, these studies will be narratively described and their potential impact on the findings will be discussed, but they will not be excluded from the review.

### Search strategy

Two researchers (ZMY, LL) will independently perform systematic searches across various databases from their inception to April 30, 2025. The search strategy will combine MeSH terms and free-text keywords related to IBS-D and acupuncture using Boolean operators (AND/OR). Two senior researchers (ZD, ZQH) will develop the strategy based on previously published studies [24, 25]. Comprehensive search strategies specific to each database are provided in Supplementary Material 2. The searches will utilize the following keywords: (1) Disease-related terms: "irritable bowel syndrome," "IBS," "IBS-D," "diarrhea-predominant irritable bowel syndrome," "irritable colon," "spastic colon," "functional bowel disease," "functional diarrhea," "visceral hypersensitivity," "gut-brain axis," "brain-gut interaction." (2) Intervention-related terms: "acupuncture," "acupuncture therapy," "manual acupuncture," "electroacupuncture," "electro-acupuncture," "moxibustion," "warm needling," "auricular acupuncture," "ear acupuncture," "acupressure," "laser acupuncture," "transcutaneous electrical acupoint stimulation," "TEAS," "acupoint," "meridian," "needle," "needling." (3) Study type and population terms: "animal," "animal model," "animal experiment," "preclinical," "rat," "rats," "mouse," "mice," "rodent," "murine," "Sprague–Dawley," "Wistar," "C57BL," "BALB/c," "mechanism," "experimental."No language filters will be applied to the electronic searches to ensure comprehensive retrieval. However, during the screening stage, studies not published in Chinese or English will be excluded (see Section “Exclusion criteria”), as the review team's linguistic expertise is limited to these two languages. The number of studies excluded at this stage on the basis of language will be documented in the PRISMA flow diagram to enable assessment of potential language bias. No publication date restrictions will be applied. Manual screening of references in relevant reviews will be performed to identify additional studies.

### Data management and study selection

**Fig. 1:**
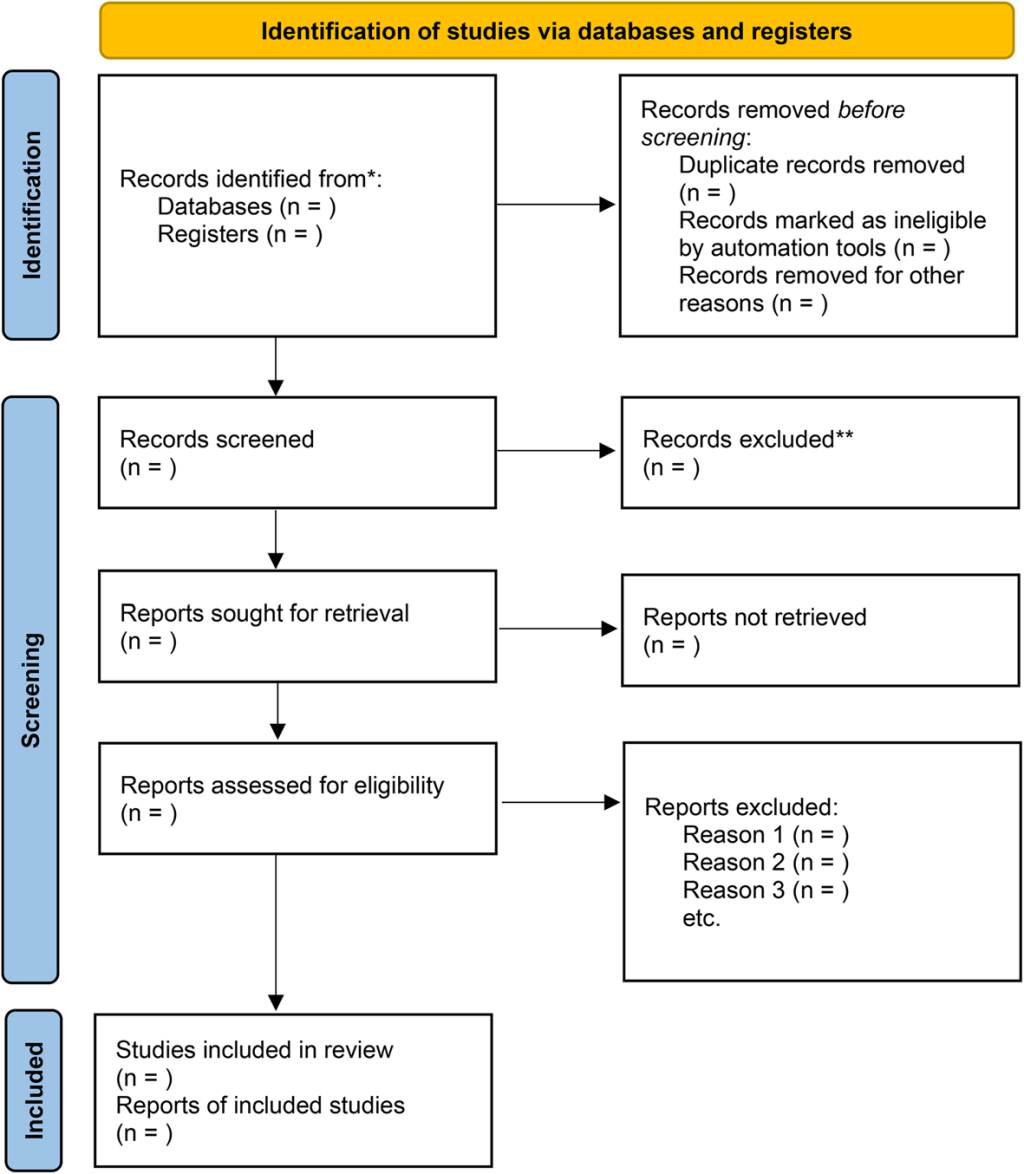
PRISMA flow chart for literature screening

All articles retrieved electronically will be imported into EndNote 21 for duplicate removal. Based on predetermined eligibility criteria, two independent reviewers (ZMY, LL) will screen studies that appear potentially relevant. During the initial screening phase, titles and abstracts will be reviewed to eliminate unrelated studies. The full texts of the remaining studies will subsequently be reviewed to determine final eligibility. Any differences will be resolved through discussion with a third reviewer (JF) [26]. Both independent reviewers (ZMY and LL) are native Chinese speakers with professional proficiency in English, ensuring accurate screening and data extraction from studies published in both Chinese and English. Bilingual proficiency will be verified through a pilot screening exercise prior to formal screening, in which both reviewers will independently screen a random sample of 50 records in each language, with discrepancies discussed and resolved to ensure consistent application of eligibility criteria. The selection process will follow the flowchart presented in Fig. 1 [27] (provided as a separate file).

### Outcome measures

The primary outcome is the minimal volume threshold for the abdominal withdrawal reflex (AWR), defined as the minimum volume of colorectal balloon distension (typically in mL) that induces a visible abdominal withdrawal reflex. This indicator is widely used in IBS-D animal models to evaluate visceral hypersensitivity [13, 19, 20] and directly reflects a core pathological feature of IBS-D.

Secondary outcomes include: (a) loose stool rate, as an objective behavioral measure of diarrhea severity that quantifies the regulatory effect of acupuncture interventions on intestinal transit function; (b) levels of inflammatory cytokines interleukin-1β (IL-1β) and tumor necrosis factor-α (TNF-α), which are key inflammation-related cytokines that exacerbate intestinal mucosal damage through the initiation of the NF-κB signaling pathway [3]. and directly reflect the immune imbalance associated with low-grade intestinal inflammation in IBS-D [4]; (c) levels of substance P (SP), a core neuropeptide mediating the transmission of visceral pain to the central nervous system, with elevated release significantly related to abdominal pain symptoms [6].; and (d) levels of serotonin (5-HT), a crucial neurotransmitter regulating gastrointestinal motility and visceral hypersensitivity [21], whose abnormal metabolism is a major driver of diarrhea and motility disturbances [22].

Combining these outcome measures allows for a comprehensive reflection of the complex pathological mechanisms of IBS-D from multiple perspectives, including visceral hypersensitivity, inflammatory response, neurotransmitter regulation, and behavioral manifestations. Studies included in the systematic review on the basis of Population, Intervention, and Comparator (PIC) criteria will contribute data to the NMA for whichever of these outcomes they report. Studies that meet PIC criteria but do not report any of the pre-specified outcomes will still be included in the systematic review to enable assessment of potential selective outcome reporting bias.

### Data extraction

Two independent researchers (ZMY, LL) will perform data extraction following a standardized protocol. A structured data extraction form will be developed based on pilot testing. Data will be independently entered into Microsoft Excel 2021 and cross-checked to ensure consistency. Discrepancies will be addressed by discussion or, if needed, adjudicated by a third researcher (JF). The extraction form will include the following items: Basic study characteristics: Author(s), study design (parallel, crossover, or sequential), and publication year. Animal characteristics: Species (e.g., SD rats, Wistar rats, C57BL/6 mice), sex (female/male/mixed/not reported), weight, age. Acupuncture details: Intervention modality (manual acupuncture/electroacupuncture/moxibustion), treatment frequency, acupoint combinations, and duration of treatment.

For the primary outcome (AWR minimal volume threshold), data will be extracted in the original units reported (typically milliliters, mL). When different units are used, appropriate conversion factors will be applied to standardize all measurements to mL. For secondary outcomes (IL-1β, TNF-α, SP, and 5-HT), data will be extracted in the units reported (e.g., pg/mL, ng/mL), and units will be documented for each study. When multiple time points are reported for the same biomarker, the measurement obtained at the earliest post-intervention time point following the conclusion of the final treatment session will be prioritized for data extraction. If only follow-up data collected at a later time point (e.g., days or weeks after the final session) are available, these will be used and the time point will be documented. All continuous variables will be standardized to the format of mean $\pm$ SD.

###  Data synthesis

All quantitative analyses will be conducted within a frequentist statistical framework using a random-effects model with restricted maximum likelihood (REML) estimation. Standardized mean differences (SMD) with 95% confidence intervals (CI) will be calculated as the primary effect measure for all continuous outcomes, including both behavioral measures (AWR threshold) and molecular biomarkers (IL-1β, TNF-α, SP, 5-HT). The use of SMD is justified because: (1) AWR measurement protocols vary across studies in terms of distension volumes, pressure increments, and balloon specifications; (2) biomarker assays differ in measurement methods (e.g., ELISA, radioimmunoassay), units (e.g., pg/mL, ng/mL, ng/L), and tissue sources (e.g., serum, colonic tissue homogenate), making direct comparison of absolute values inappropriate. SMD standardizes these differences by expressing each study's effect in terms of its own standard deviation, enabling valid cross-study comparisons. When a subset of studies uses identical measurement methods and units for a specific biomarker, a sensitivity analysis using weighted mean difference (WMD) will be conducted as a supplementary analysis to provide absolute effect estimates in the original units. If raw data are presented only in graphical form, values will be extracted using GetData Graph Digitizer 2.26. Standard deviations (SD) will be derived using standard formulas (e.g.,${\boldsymbol{S}}{\boldsymbol{D}}={\boldsymbol{S}}{\boldsymbol{E}}{\boldsymbol{M}}\times \sqrt{{\boldsymbol{n}}}$) where necessary. Study authors may also be contacted to obtain missing data values.

For multi-arm trials involving more than two intervention groups, the within-study correlation structure will be preserved by utilizing a multivariate meta-analysis approach. Specifically, all pairwise comparisons from multi-arm studies will be included simultaneously in the network, and the correlation between treatment effects will be accounted for by using the within-study variance–covariance matrix. This approach preserves randomization and avoids double-counting of shared control groups. Studies comparing multiple acupuncture modalities, for example, manual acupuncture versus electroacupuncture versus moxibustion versus control, will thus contribute multiple treatment contrasts to the network while maintaining appropriate statistical dependencies.

Heterogeneity will be assessed at two levels. First, for each pairwise comparison within the network, heterogeneity will be quantified using the *I*^*2*^ statistic and the Cochran's Q test. Second, global heterogeneity across the entire network will be evaluated using the between-study variance parameter (τ^2^) estimated under the random-effects model in netmeta, along with the generalized Q statistic for the overall network. The decomposition of Q into within-design and between-design components will be examined to further understand the sources of heterogeneity and to detect potential inconsistency (see Section “Transitivity assessment” for details). If *I*^*2*^ > 50%, factors contributing to heterogeneity will be identified (e.g., animal strain, treatment duration) by conducting meta-regression. Statistical analyses and graphical representations will be performed utilizing R 4.4.1 and Stata 17.0.

For secondary outcomes involving biomarkers within the same category (e.g., inflammatory cytokines IL-1β and TNF-α), each biomarker will be analyzed separately in the NMA whenever three or more studies reporting that specific biomarker are available. When fewer than three studies report a specific biomarker, precluding a meaningful quantitative analysis, the findings for that biomarker will be narratively synthesized. In the event that separate analyses for individual biomarkers within the same category yield qualitatively similar results, a pooled analysis combining biomarkers within that category may be conducted as a supplementary sensitivity analysis, with the rationale and approach transparently reported.

For networks containing closed loops, local inconsistency between direct and indirect evidence will be evaluated using the node-splitting approach [28]. This method separates the evidence for each treatment comparison into its direct and indirect components and tests whether these two sources of evidence are in statistical agreement. A p-value < 0.05 from the node-splitting test will be considered indicative of significant local inconsistency for that particular comparison. Additionally, global inconsistency across the entire network will be assessed using the design-by-treatment interaction model, which tests whether the treatment effects estimated from different study designs within the network are consistent. A net heat plot will be generated to visually identify specific comparisons or designs that contribute most to inconsistency in the network. If significant inconsistency is detected, the following steps will be taken: (1) the potential clinical or methodological sources of inconsistency will be explored (e.g., differences in control group types, animal strains, or modeling methods); (2) sensitivity analyses will be conducted by excluding studies that may be driving the inconsistency; and (3) if inconsistency persists, results from both the consistency and inconsistency models will be reported and findings will be interpreted with appropriate caution; (4) the certainty of evidence for affected comparisons will be downgraded.

Treatment ranking probabilities will be computed using P-scores, the frequentist analogue of SUCRA (surface under the cumulative ranking curve). P-scores will be calculated using the netmeta package in R. P-scores range from 0 to 100%, with higher values indicating greater probability of a treatment being among the best options [29]. Treatments with P-score greater than 80% will be considered high-ranking, values between 50 and 80% will indicate moderate-ranking, and values less than 50% will suggest low-ranking treatments. The uncertainty in rankings will be visualized using rankograms showing the probability distribution of each treatment across all possible ranks.

The clinical relevance of effect sizes will be interpreted based on Cohen's conventions: SMD values between 0.2 and 0.5 signify a small effect; between 0.5 and 0.8, a moderate effect; and above 0.8, a large effect [30]. To ensure full reproducibility and transparency, all analysis code including R and Stata scripts, dataset structure templates, and detailed package version information will be publicly archived on the Open Science Framework repository upon completion of the review. The Open Science Framework repository link will be provided in the final published systematic review. Key R packages to be used include netmeta (version ≥ 2.9–0) for frequentist network meta-analysis, meta for conventional pairwise meta-analyses, and ggplot2 for visualization. Stata commands will include network meta, mvmeta, and related graphical functions.

#### Definition of treatment nodes

Treatment nodes in the network meta-analysis will be defined based on the primary acupuncture modality. The main nodes are: (a) manual acupuncture (MA), (b) electroacupuncture (EA), (c) moxibustion (Mox), (d) other acupuncture therapies (e.g., auricular acupuncture, warm needling), (e) sham acupuncture, (f) pharmacological control, and (g) model/blank control.

In the primary analysis, all electroacupuncture studies will be pooled into a single EA node regardless of stimulation parameters (frequency, intensity, waveform), and all moxibustion studies will be pooled into a single Mox node regardless of moxibustion type (mild moxibustion, herb-partitioned moxibustion, heat-sensitive moxibustion). This pooling strategy is justified by the need to maintain sufficient studies per node for meaningful network comparisons, as excessive node splitting would likely result in a sparse network with unreliable estimates. To explore potential effect modification by specific parameters, the following pre-planned sensitivity/subgroup analyses will be conducted if sufficient data (≥ 3 studies per subnode) are available: (1) EA frequency: 2 Hz (low frequency) vs. 100 Hz (high frequency) vs. 2/100 Hz alternating frequency as separate nodes;(2) Moxibustion type: mild moxibustion vs. herb-partitioned moxibustion vs. heat-sensitive moxibustion as separate nodes. (3) Acupoint combinations: single acupoint vs. multi-acupoint prescriptions.

If data are insufficient for formal subgroup NMA with split nodes, these parameters will be explored via meta-regression (see Section “Meta-regression”). The decision to pool or split nodes will be transparently reported, along with the number of studies contributing to each node, in the final systematic review.

#### Analysis hierarchy and data contingency plans

The primary analysis will be the frequentist random-effects NMA for the primary outcome (AWR threshold) using pooled treatment nodes. The following are designated as pre-planned secondary analyses: NMA for each secondary outcome (loose stool rate, IL-1β, TNF-α, SP, 5-HT). The following are designated as pre-planned exploratory analyses: (a) subgroup NMA with split treatment nodes (e.g., EA by frequency); (b) meta-regression for effect modifier identification; (c) sensitivity analyses (leave-one-out, risk of bias-based exclusion). Several planned analyses are contingent on the availability of sufficient data. The following minimum data requirements and contingency plans are specified: Network meta-analysis: A minimum of three studies per treatment node is required for inclusion in the network. If a treatment node has fewer than three studies, it will be excluded from the NMA but will be described narratively. Meta-regression: A minimum of ten studies per comparison is required (see Section “Meta-regression”). If this threshold is not met, meta-regression will not be performed, and potential effect modifiers will be discussed qualitatively. Subgroup analysis: A minimum of three studies per subgroup is required (see Section “Subgroup analysis”). If insufficient data are available for a pre-specified subgroup, this limitation will be reported. Node-split sensitivity analyses (separate EA frequency or moxibustion type nodes): A minimum of three studies per subnode is required. If data are insufficient, parameters will be explored via meta-regression or narrative synthesis. Publication bias assessment: Conventional funnel plots and Egger's test require a minimum of ten studies per comparison.

If the overall data availability proves severely limited, the primary analysis will prioritize: (1) the primary outcome (AWR threshold), (2) the most commonly reported secondary outcomes, and (3) pairwise meta-analyses where network meta-analysis is not feasible. All deviations from the planned analyses due to data limitations will be transparently documented and justified in the final systematic review.

### Risk of bias assessment

Two researchers (ZMY, LL) will independently assess bias using the SYRCLE risk-of-bias tool [31, 32]. This tool evaluates ten signaling questions across six domains. The first domain addresses selection bias through assessment of sequence generation, baseline characteristics, and allocation concealment. The second domain evaluates performance bias by examining random housing and blinding of caregivers and investigators. The third domain concerns detection bias, referring to random outcome assessment and blinding of outcome assessors. The fourth domain addresses attrition bias, which concerns incomplete outcome data and intention-to-treat analysis. The fifth domain evaluates reporting bias related to selective outcome reporting. The sixth domain covers other sources of bias including conflicts of interest, funding sources, and other potential biases.

Each signaling question will be rated as "Yes" indicating low risk of bias, "No" indicating high risk of bias, or "Unclear" when there is insufficient information to make a judgment. Studies will be categorized as having low, unclear, or high overall risk of bias based on domain-level assessments. Any disagreements between the two reviewers (ZMY, LL) will be resolved through discussion with a third experienced researcher (JF), who will make the final decision.

### Heterogeneity assessment

Given the anticipated heterogeneity in abdominal withdrawal reflex measurement protocols, including varying distension volumes, pressure increments, and balloon types, standardized mean differences will be used as the primary effect size metric to enable comparisons across studies using different measurement scales [28]. Heterogeneity will be classified as follows: I^2^ of 0% to 40%: low heterogeneity; I^2^ of 30% to 50%: moderate heterogeneity; I^2^ of 50% to 75%: substantial heterogeneity; I^2^ of 75% to 100%: considerable heterogeneity [33]. For *I*^*2*^ above 50%, subgroup analyses (e.g., by modeling method, intervention frequency, animal species) and meta-regression will be performed to explore potential sources of heterogeneity. In meta-regression analyses, a significance threshold of P < 0.10 will be used to identify covariates that may explain heterogeneity, as this more liberal threshold is commonly adopted in exploratory meta-regression to avoid overlooking potentially important effect modifiers given the typically limited statistical power of such analyses. Covariates identified at P < 0.10 in univariate meta-regression will be considered candidates for multivariate meta-regression if three or more covariates are significant (see Section “Meta-regression” for details).

Sensitivity analysis using the leave-one-out (LOO) method will assess the impact of individual studies by iteratively excluding each study and comparing changes in pooled effect sizes, CIs, and heterogeneity metrics (e.g. I^2^). For analyses exhibiting substantial heterogeneity under a random-effects model, restricted maximum likelihood (REML) estimation will be used as the default method for estimating the between-study variance (τ^2^). If REML produces boundary estimates (e.g., τ^2^ = 0), alternative estimators such as the DerSimonian-Laird or Paule-Mandel methods will be applied as sensitivity analyses to assess the robustness of variance estimates. All analyses will default to two-tailed tests, with P < 0.05 considered statistically significant, though results will be interpreted alongside effect sizes and CIs. For substantial heterogeneity (I^2^ > 50% or P < 0.05), meta-regression, subgroup analysis, and sensitivity analysis will be employed to explore potential influencing factors [34].

### Reporting bias assessment

Publication bias will be assessed using both pairwise and network-specific methods to ensure comprehensive evaluation across the treatment network. For pairwise comparisons, when treatment comparisons include ten or more studies, conventional funnel plots will be generated by plotting effect sizes against their standard errors. Asymmetry will be tested using Egger's regression test or Begg's rank correlation test, with a P-value less than 0.05 indicating significant bias.

For network meta-analysis-specific methods, comparison-adjusted funnel plots will be constructed to detect small-study effects across the entire treatment network. These plots will display comparison-adjusted effect sizes against their standard errors, with asymmetry suggesting potential publication bias or systematic differences between small and large studies. Additionally, network meta-regression will be performed to test whether effect sizes are associated with study precision, using standard error as a covariate to provide a formal test for small-study effects within the network structure.

If significant publication bias is detected through asymmetric comparison-adjusted funnel plots or P-values less than 0.05 in network meta-regression, the following measures will be implemented. First, sensitivity analyses excluding small studies (defined as those with fewer than ten animals per group) will be conducted. Second, trim-and-fill methods will be applied where applicable to adjust for potential missing studies. Third, the potential impact on effect estimates and certainty of evidence will be explicitly discussed. The results will be interpreted with appropriate caution, and limitations will be transparently reported in the final review [35].

### Transitivity assessment

Assessing transitivity is crucial for the validity of network meta-analysis. The netmeta package in R 4.4.1 will be used to generate network evidence plots, and the Weighted Node Degree will be calculated to evaluate the strength of study connections.

Beyond statistical evaluation using node-splitting analysis, potential transitivity violations will be addressed through the following steps: (1) If significant inconsistency (P < 0.05) is detected and attributed to differences in control groups (e.g., sham acupuncture vs. no treatment), separate network meta-analyses will be conducted by control group type when sufficient data are available. (2) If violations are associated with key effect modifiers (e.g., animal species, modeling method), subgroup analyses will be performed using studies with comparable characteristics. (3) For unresolved violations, the certainty of evidence will be downgraded, and the limitations will be explicitly discussed. This multi-step approach will help ensure the validity of indirect comparisons in the network meta-analysis.

### Meta-regression

Meta-regression will be performed to explore sources of heterogeneity and potential effect modifiers when sufficient studies, defined as ten or more studies per comparison, are available. The following covariates will be pre-specified for meta-regression analyses to minimize the risk of exploratory bias and ensure transparent reporting.

Animal-related factors to be examined include animal species comparing rats versus mice, animal strain such as Sprague–Dawley rats versus Wistar rats or C57BL/6 mice versus BALB/c mice, sex categorized as male, female, mixed, or not reported, and baseline weight or age at intervention initiation. IBS-D modeling-related factors include modeling method categorized as chemical induction using agents such as acetic acid or senna, stress-based models including maternal separation or restraint stress, or post-infection models, as well as the duration from model establishment to intervention initiation.

Intervention-related factors encompass treatment duration classified as short-term defined as fourteen days or less, medium-term from fifteen to twenty-eight days, or long-term exceeding twenty-eight days. Treatment frequency will be examined comparing daily versus alternate days versus other schedules. For electroacupuncture specifically, stimulation frequency will be analyzed comparing 2 Hz versus 100 Hz versus alternating frequencies. For moxibustion, treatment duration per session will be compared between sessions lasting less than fifteen minutes versus fifteen minutes or longer.

Study quality factors to be considered include sample size per group comparing fewer than ten animals versus ten or more animals, and risk of bias level categorized as low, unclear, or high based on SYRCLE assessment results. These covariates will be tested individually in univariate meta-regression models first. If three or more covariates show significant associations with a P-value threshold of less than 0.10, multivariate meta-regression will be performed using backward elimination to identify independent predictors. Results will be interpreted considering potential collinearity between variables and the implications of multiple testing, with appropriate adjustments made when necessary. If the minimum threshold of ten studies per comparison is not met, meta-regression will not be performed, and potential effect modifiers will be discussed qualitatively.

### Subgroup analysis

Subgroup analyses will be conducted for pre-specified stratification variables when sufficient data are available, defined as three or more studies per subgroup, to ensure meaningful comparisons. These subgroup analyses will help identify potential sources of heterogeneity and explore whether treatment effects vary across different study characteristics.

For IBS-D modeling method, studies will be grouped into chemical induction using acetic acid, senna, or other chemical irritants, stress-based models including maternal separation, restraint stress, or water avoidance stress, and post-infection models. Animal species and strain will be examined by comparing rats including Sprague–Dawley, Wistar, and others versus mice including C57BL/6, BALB/c, and others. Treatment duration will be categorized into short-term defined as fourteen days or less, medium-term from fifteen to twenty-eight days, and long-term exceeding twenty-eight days. Control group type will be stratified into sham acupuncture control, blank control with no treatment, model control, and active pharmacological control.

Subgroup differences will be assessed using meta-regression or Chi-squared tests, with P-values less than 0.05 indicating significant subgroup effects. If substantial subgroup effects are identified, suggesting meaningful differences in treatment effects across subgroups, separate network meta-analyses may be performed for distinct subgroups where feasible. This approach will provide more precise estimates for specific experimental scenarios and help guide the interpretation of results for different experimental contexts. If insufficient data are available for a pre-specified subgroup, this limitation will be reported and the subgroup will be described narratively.

### Sensitivity analysis

Sensitivity analyses will be performed to assess the robustness of the primary findings and to determine whether conclusions are sensitive to methodological decisions or the inclusion of particular studies. Multiple sensitivity analyses are planned to comprehensively evaluate result stability.

First, leave-one-out analysis will be conducted where each study will be iteratively excluded from the network, and the pooled effect sizes, confidence intervals, and heterogeneity metrics such as *I*^*2*^ will be recalculated to identify influential studies that may disproportionately affect the overall results. Second, risk of bias-based sensitivity analysis will be performed where feasible, meaning when sufficient studies remain after exclusion. Studies rated as high risk of bias in three or more SYRCLE domains will be excluded, and the network meta-analysis will be repeated using only studies with low or unclear risk of bias. The consistency of results between the full network and the sensitivity analysis restricted to lower risk studies will be compared to determine whether study quality substantially affects the conclusions.

Third, heterogeneity-driven sensitivity analysis will be implemented. If high heterogeneity defined as *I*^*2*^ greater than 50% is detected, the trim-and-fill method will be applied to assess the potential impact of outlier studies or potential publication bias on the pooled estimates. Changes in effect size magnitude, direction, statistical significance, and treatment rankings as measured by P-scores values will be documented and reported. If sensitivity analyses yield substantially different results from the primary analysis, conclusions will be appropriately qualified, and limitations will be discussed in detail to ensure that readers understand the uncertainty surrounding the findings.

## Discussion

This study will employ network meta-analysis to effectively integrate basic research data on acupuncture for IBS-D animal models. This approach significantly increases the sample size, reduces random error from individual studies [36, 37], and systematically explores the efficacy and related biological mechanisms of acupuncture therapies in IBS-D animal models. By publishing a detailed protocol, we aim to enhance the openness and transparency of our research objectives and methods.

## Research significance

Although acupuncture has shown significant potential in relieving the core symptoms of IBS-D (e.g., diarrhea, abdominal pain) and has accumulated a wealth of preclinical evidence [10, 11], there is still a major gap in comparative efficacy research. Existing animal experiments are usually limited to comparing a single acupuncture therapy (e.g., electroacupuncture or moxibustion) with a placebo [37], lacking a systematic efficacy evaluation across different therapies. This study is the first to integrate direct and indirect evidence and systematically compare the efficacy differences of different acupuncture therapies (manual acupuncture, electroacupuncture, moxibustion, etc.) in an IBS-D animal model. The aim is to determine the best intervention (e.g., electroacupuncture regulates visceral hypersensitivity, moxibustion inhibits intestinal inflammation) and provide experimental evidence for the development of standardized clinical acupuncture protocols.

The high controllability of animal models provides a unique advantage for elucidating the mechanism of action of acupuncture. By measuring multidimensional indicators such as IL-1β, TNF-α (inflammatory pathway), SP, 5-HT (neural regulation), and loose stool rate (behavioral phenotype), this study can summarize and integrate the biological mechanisms of the efficacy of different acupuncture therapies. For example, electroacupuncture may regulate aquaporins by inhibiting inflammatory signaling pathways, thereby restoring the balance of water metabolism and intestinal permeability in IBS-D models [38]. Moxibustion may alleviate visceral hypersensitivity in patients with IBS-D by reducing the number of mast cells and inhibiting the SCF/c-kit signaling pathway [39]. Due to ethical restrictions, these mechanisms are difficult to explore in depth in clinical studies, and animal experiments provide an irreplaceable method for revealing the signaling pathways of acupuncture regulating the "brain-gut axis". Furthermore, through subgroup analysis, this study will explore the influence of modeling methods (e.g., maternal separation vs. acetic acid enema) and treatment duration (short-term vs. long-term) on efficacy. This will provide critical references for designing future animal experiments and promote the translation of acupuncture research from basic science to clinical application.

Animal models offer an excellent platform for studying the pathophysiological mechanisms of IBS-D, allowing researchers to distinguish different signaling pathways and investigate how acupuncture modulates them. Currently, the exact pathogenesis of IBS-D is unclear, but it may involve mechanisms such as chronic low-grade intestinal inflammation, the brain-gut axis, visceral hypersensitivity, and gastrointestinal motility dysfunction, as well as biomarkers such as SP, 5-HT, IL-1β, and TNF-α. This study will systematically compare different acupuncture therapies using a network meta-analysis, aiming to elucidate the effects of different acupuncture methods on different biomarkers and their signaling pathways, thereby revealing the relationship between different acupuncture therapies and these biomarkers. In clinical studies, it is often challenging to study these biomarkers and signaling pathways in humans due to practical and ethical limitations.

### Strengths

This study presents several key strengths that enhance the robustness and relevance of its findings. Firstly, it is the first to apply NMA to compare multiple acupuncture therapies (e.g., electroacupuncture, moxibustion, and auricular acupuncture) in animal models of IBS-D. By integrating both direct and indirect comparisons, NMA overcomes the limitations of conventional single-modality analyses and ranks treatment efficacy using P-scores. Secondly, the study conducts a dual assessment of symptoms and mechanisms, evaluating both behavioral outcomes (AWR threshold, loose stool rate) and molecular biomarkers (TNF-α, SP, IL-1β, and 5-HT) to provide a multidimensional understanding of therapeutic effects. For instance, AWR thresholds reflect improvements in visceral hypersensitivity, while 5-HT levels indicate regulation of colonic motility [40]. Finally, subgroup analysis and meta-regression explore the influence of modeling methods, animal strains, and treatment duration on efficacy, offering targeted insights for future IBS-D animal experiments.

### Limitations

This study has several limitations that should be acknowledged. First, inconsistent modeling standards across the included studies, such as the use of diarrhea scores in some and inflammatory markers in others, may introduce selection bias. Future studies should aim to develop a unified evaluation system for IBS-D animal models that combines behavioral and pathological indicators. Second, the lack of standardization in acupuncture-related parameters, such as electroacupuncture frequency and moxibustion dosage, may affect the comparability of treatment efficacy across studies. Establishing consistent intervention protocols is necessary to ensure more reliable comparisons. Third, findings derived from rodent models must be interpreted with caution when extrapolating to human IBS-D. Different animal species (rats vs. mice) may exhibit distinct physiological responses to both IBS-D induction and acupuncture interventions. Similarly, different model induction methods (e.g., chemical irritation vs. psychological stress) may activate different pathophysiological pathways, potentially influencing the apparent efficacy of acupuncture therapies. These translational limitations will be carefully considered when interpreting the results. The planned subgroup analyses by animal species and modeling method (Section “Subgroup analysis”) will help quantify the extent to which these factors influence treatment effect. Fourth, animal models do not fully replicate the complexity of human IBS-D, particularly regarding comorbid conditions (e.g., anxiety and depression) and individual variations in treatment response. Multi-center RCTs are needed to validate and generalize the observed efficacy outcomes.

## Supplementary Information

Supplementary Material 1.

Supplementary Material 2.

## Data Availability

The review’s protocol is available at INPLASY (INPLASY202470113).

## Abbreviations

AGA: American Gastroenterological Association
CI: Confidence interval
IBS-D: Diarrhea-predominant irritable bowel syndrome
NMA: Network meta-analysis
P-score: Frequentist analogue of surface under the cumulative ranking curve;SUCRAsurface under the cumulative ranking curve
SMD: Standardized mean difference
AWR: Abdominal withdrawal reflex
IL-1β: Interleukin-1β
TNF-α: Tumor necrosis factor-α
SP: Substance P
5-HT: Serotonin, REMLrestricted maximum likelihood
WMD: Weighted mean difference
PIC: Population, intervention, comparator
PICO: Population, intervention, comparator, outcome
