# Supplementary material for: Comparative effectiveness and underlying mechanisms of acupuncture approaches in IBS-D animal models: a protocol for systematic review and network meta-analysis
Source: Syst Rev. 2026 Mar 26;15:152. doi: 10.1186/s13643-026-03162-5 (PMC13141318; doi:10.1186/s13643-026-03162-5)
Supplement: Supplementary file 2 — Supplementary Material 2. [file 13643_2026_3162_MOESM2_ESM.docx]

**Appendix 1**

Search strategy for pubmed database

| No. | Search |
| --- | --- |
| #1 | ("Irritable Bowel Syndrome"[MeSH Terms] OR "irritable bowel syndrome"[Title/Abstract] OR "IBS"[Title/Abstract] OR "IBS-D"[Title/Abstract] OR "diarrhea-predominant irritable bowel syndrome"[Title/Abstract] OR "irritable colon"[Title/Abstract] OR "spastic colon"[Title/Abstract] OR "functional bowel disease"[Title/Abstract] OR "functional diarrhea"[Title/Abstract] OR "visceral hypersensitivity"[Title/Abstract] OR "gut-brain axis"[Title/Abstract] OR "brain-gut interaction"[Title/Abstract]) |
| #2 | ("Acupuncture Therapy"[MeSH Terms] OR "acupuncture"[Title/Abstract] OR "acupuncture therapy"[Title/Abstract] OR "manual acupuncture"[Title/Abstract] OR "electroacupuncture"[Title/Abstract] OR "electro-acupuncture"[Title/Abstract] OR "moxibustion"[Title/Abstract] OR "warm needling"[Title/Abstract] OR "auricular acupuncture"[Title/Abstract] OR "ear acupuncture"[Title/Abstract] OR "acupressure"[Title/Abstract] OR "laser acupuncture"[Title/Abstract] OR "transcutaneous electrical acupoint stimulation"[Title/Abstract] OR "TEAS"[Title/Abstract] OR "acupoint"[Title/Abstract] OR "meridian"[Title/Abstract] OR "needle"[Title/Abstract] OR "needling"[Title/Abstract]) |
| #3 | ("Animals"[MeSH Terms] OR "animal model"[Title/Abstract] OR "animal experiment"[Title/Abstract] OR "preclinical"[Title/Abstract] OR "rat"[Title/Abstract] OR "rats"[Title/Abstract] OR "mouse"[Title/Abstract] OR "mice"[Title/Abstract] OR "rodent*"[Title/Abstract] OR "murine"[Title/Abstract] OR "Sprague-Dawley"[Title/Abstract] OR "Wistar"[Title/Abstract] OR "C57BL"[Title/Abstract] OR "BALB/c"[Title/Abstract]) |
| #4 | ("mechanism"[Title/Abstract] OR "mechanisms"[Title/Abstract] OR "experimental"[Title/Abstract]) |
| #5 | #3 OR #4 |
| #6 | #1 AND #2 AND #5 |

Search strategy for Embase database

| No. | Search |
| --- | --- |
| #1 | ('irritable bowel syndrome'/exp OR 'irritable bowel syndrome':ti,ab OR 'IBS':ti,ab OR 'IBS-D':ti,ab OR 'diarrhea predominant irritable bowel syndrome':ti,ab OR 'irritable colon':ti,ab OR 'spastic colon':ti,ab OR 'functional bowel disease':ti,ab OR 'functional diarrhea':ti,ab OR 'visceral hypersensitivity':ti,ab OR 'gut brain axis':ti,ab OR 'brain gut interaction':ti,ab) |
| #2 | ('acupuncture'/exp OR 'acupuncture therapy'/exp OR 'acupuncture':ti,ab OR 'acupuncture therapy':ti,ab OR 'manual acupuncture':ti,ab OR 'electroacupuncture':ti,ab OR 'electro-acupuncture':ti,ab OR 'moxibustion'/exp OR 'moxibustion':ti,ab OR 'warm needling':ti,ab OR 'auricular acupuncture':ti,ab OR 'ear acupuncture':ti,ab OR 'acupressure':ti,ab OR 'laser acupuncture':ti,ab OR 'transcutaneous electrical acupoint stimulation':ti,ab OR 'TEAS':ti,ab OR 'acupoint':ti,ab OR 'meridian':ti,ab OR 'needle':ti,ab OR 'needling':ti,ab) |
| #3 | ('animal'/exp OR 'animal model':ti,ab OR 'animal experiment':ti,ab OR 'preclinical':ti,ab OR 'rat'/exp OR 'rat':ti,ab OR 'rats':ti,ab OR 'mouse'/exp OR 'mouse':ti,ab OR 'mice':ti,ab OR 'rodent*':ti,ab OR 'murine':ti,ab OR 'Sprague-Dawley':ti,ab OR 'Wistar':ti,ab OR 'C57BL':ti,ab OR 'BALB/c':ti,ab) |
| #4 | ('mechanism':ti,ab OR 'mechanisms':ti,ab OR 'experimental':ti,ab) |
| #5 | #3 OR #4 |
| #6 | #1 AND #2 AND #5 |

Search strategy for Cochrane Library database

| No. | Search |
| --- | --- |
| #1 | MeSH descriptor: [Irritable Bowel Syndrome] explode all trees |
| #2 | ("irritable bowel syndrome" OR IBS OR IBS-D OR "diarrhea-predominant irritable bowel syndrome" OR "irritable colon" OR "spastic colon" OR "functional bowel disease" OR "functional diarrhea" OR "visceral hypersensitivity" OR "gut-brain axis" OR "brain-gut interaction"):ti,ab,kw |
| #3 | #1 OR #2 |
| #4 | MeSH descriptor: [Acupuncture Therapy] explode all trees |
| #5 | MeSH descriptor: [Moxibustion] explode all trees |
| #6 | MeSH descriptor: [Acupressure] explode all trees |
| #7 | ("acupuncture" OR "acupuncture therapy" OR "manual acupuncture" OR electroacupuncture OR "electro-acupuncture" OR moxibustion OR "warm needling" OR "auricular acupuncture" OR "ear acupuncture" OR acupressure OR "laser acupuncture" OR "transcutaneous electrical acupoint stimulation" OR TEAS OR acupoint* OR meridian* OR needle OR needling):ti,ab,kw |
| #8 | #4 OR #5 OR #6 OR #7 |
| #9 | (animal OR "animal model" OR "animal experiment" OR preclinical OR rat OR rats OR mouse OR mice OR rodent OR murine OR "Sprague-Dawley" OR Wistar OR C57BL OR "BALB/c" OR mechanism OR experimental):ti,ab,kw |
| #10 | #3 AND #8 AND #9 |

Search strategy for Web of Science database

| No. | Search |
| --- | --- |
| #1 | TS=("irritable bowel syndrome" OR IBS OR "IBS-D" OR "diarrhea-predominant irritable bowel syndrome" OR "irritable colon" OR "spastic colon" OR "functional bowel disease" OR "functional diarrhea" OR "visceral hypersensitivity" OR "gut-brain axis" OR "brain-gut interaction") |
| #2 | TS=("acupuncture" OR "acupuncture therapy" OR "manual acupuncture" OR electroacupuncture OR "electro-acupuncture" OR moxibustion OR "warm needling" OR "auricular acupuncture" OR "ear acupuncture" OR acupressure OR "laser acupuncture" OR "transcutaneous electrical acupoint stimulation" OR TEAS OR acupoint* OR meridian* OR needle OR needling) |
| #3 | TS=("animal" OR "animal model" OR "animal experiment" OR preclinical OR rat OR rats OR mouse OR mice OR rodent OR murine OR "Sprague-Dawley" OR Wistar OR C57BL OR "BALB/c" OR mechanism OR experimental) |
| #4 | #1 AND #2 AND #3 |

**Search strategy of CNKI**

| No. | Search |
| --- | --- |
| #1 | SU=('肠易激综合征'+'腹泻型肠易激综合征'+'腹泻为主型肠易激综合征'+'肠易激'+'功能性腹泻'+'内脏高敏感'+'内脏高敏'+'肠-脑轴'+'脑-肠互动'+'脑肠互动'+'脑肠轴'+'IBS'+'IBS-D') |
| #2 | SU=('针刺'+'针灸'+'针刺疗法'+'针灸疗法'+'手针'+'电针'+'电针疗法'+'艾灸'+'温针'+'耳针'+'耳穴针刺'+'耳穴疗法'+'耳穴压豆'+'耳穴压迫'+'穴位按压'+'指压'+'穴位按摩'+'激光针灸'+'经皮穴位电刺激'+'经皮电穴位刺激'+'经皮穴位电刺激疗法'+'TEAS'+'穴位'+'经络'+'针'+'针刺操作'+'针刺法') |
| #3 | SU=('动物'+'动物模型'+'动物实验'+'实验动物'+'前临床'+'大鼠'+'小鼠'+'啮齿类'+'鼠类'+'rat'+'rats'+'mouse'+'mice'+'rodent'+'murine'+'Sprague-Dawley'+'Wistar'+'C57BL'+'BALB/c') |
| #4 | SU=('机制'+'机制研究'+'实验性'+'实验研究'+'experimental') |
| #5 | #3+#4 |
| #6 | #1*#2*#5 |

**Search strategy of Wanfang Data**

| No. | Search |
| --- | --- |
| #1 | 主题:('肠易激综合征'+'腹泻型肠易激综合征'+'腹泻为主型肠易激综合征'+'肠易激'+'功能性腹泻'+'内脏高敏感'+'内脏高敏'+'肠-脑轴'+'脑-肠互动'+'脑肠互动'+'脑肠轴'+'IBS'+'IBS-D') |
| #2 | 主题:('针刺'+'针灸'+'针刺疗法'+'针灸疗法'+'手针'+'电针'+'电针疗法'+'艾灸'+'温针'+'耳针'+'耳穴针刺'+'耳穴疗法'+'耳穴压豆'+'耳穴压迫'+'穴位按压'+'指压'+'激光针灸'+'经皮穴位电刺激'+'经皮电穴位刺激'+'经皮穴位电刺激疗法'+'TEAS') |
| #3 | 主题:('动物'+'动物模型'+'动物实验'+'实验动物'+'前临床'+'大鼠'+'小鼠'+'啮齿类'+'鼠类'+'rat'+'rats'+'mouse'+'mice'+'rodent'+'murine'+'Sprague-Dawley'+'Wistar'+'C57BL'+'BALB/c') |
| #4 | 主题:('机制'+'机制研究'+'实验性'+'实验研究'+'experimental') |
| #5 | #3+#4 |
| #6 | #1*#2*#5 |

**Search strategy of VIP**

| No. | Search |
| --- | --- |
| #1 | 题名或关键词:('肠易激综合征'+'腹泻型肠易激综合征'+'腹泻为主型肠易激综合征'+'肠易激'+'功能性腹泻'+'内脏高敏感'+'内脏高敏'+'肠-脑轴'+'脑-肠互动'+'脑肠互动'+'脑肠轴'+'IBS'+'IBS-D') |
| #2 | 题名或关键词:('针刺'+'针灸'+'针刺疗法'+'针灸疗法'+'手针'+'电针'+'电针疗法'+'艾灸'+'温针'+'耳针'+'耳穴针刺'+'耳穴疗法'+'耳穴压豆'+'耳穴压迫'+'穴位按压'+'指压'+'激光针灸'+'经皮穴位电刺激'+'经皮电穴位刺激'+'经皮穴位电刺激疗法'+'TEAS') |
| #3 | 题名或关键词:('动物'+'动物模型'+'动物实验'+'实验动物'+'前临床'+'大鼠'+'小鼠'+'啮齿类'+'鼠类'+'rat'+'rats'+'mouse'+'mice'+'rodent'+'murine'+'Sprague-Dawley'+'Wistar'+'C57BL'+'BALB/c') |
| #4 | 题名或关键词:('机制'+'机制研究'+'实验性'+'实验研究'+'experimental') |
| #5 | #3+#4 |
| #6 | #1*#2*#5 |

**Search strategy of SinoMed**

| No. | Search |
| --- | --- |
| #1 | 常用字段:("肠易激综合征" OR "腹泻型肠易激综合征" OR "腹泻为主型肠易激综合征" OR "肠易激" OR "功能性腹泻" OR "内脏高敏感" OR "内脏高敏" OR "肠-脑轴" OR "脑-肠互动" OR "脑肠互动" OR "脑肠轴" OR "IBS" OR "IBS-D") |
| #2 | 常用字段:("针刺" OR "针灸" OR "针刺疗法" OR "针灸疗法" OR "手针" OR "电针" OR "电针疗法" OR "艾灸" OR "温针" OR "耳针" OR "耳穴针刺" OR "耳穴疗法" OR "耳穴压豆" OR "耳穴压迫" OR "穴位按压" OR "指压" OR "激光针灸" OR "经皮穴位电刺激" OR "经皮电穴位刺激" OR "经皮穴位电刺激疗法" OR "TEAS") |
| #3 | 常用字段:("动物" OR "动物模型" OR "动物实验" OR "实验动物" OR "前临床" OR "大鼠" OR "小鼠" OR "啮齿类" OR "鼠类" OR "rat" OR "rats" OR "mouse" OR "mice" OR "rodent" OR "murine" OR "Sprague-Dawley" OR "Wistar" OR "C57BL" OR "BALB/c") |
| #4 | 常用字段:("机制" OR "机制研究" OR "实验性" OR "实验研究" OR "experimental") |
| #5 | #3 OR #4 |
| #6 | #1 AND #2 AND #5 |
